# Mental Health problems and psychotherapy in female victims of human trafficking

**DOI:** 10.1192/j.eurpsy.2023.2395

**Published:** 2023-07-19

**Authors:** F. S. Ali

**Affiliations:** Psychiatry, BorgElArab Central Hospital, Alexandria, Egypt

## Abstract

**Introduction:**

Human trafficking is the trade of humans for the purpose of forced labor, sexual slavery, or commercial sexual exploitation for the trafficker or others. This may encompass providing a spouse in the context of forced marriage or the extraction of organs.

Research into the mental health impact of trafficking has consistently found high rates of mental health problems, most commonly depression, anxiety, and posttraumatic stress disorder (PTSD).

While there is some evidence that these factors can contribute the diagnosis of PTSD, exposure to trauma is the most important feature in the development of PTSD.

Other Mental Health Problems in victims of

Trafficking:

1. Dissociative disorders
2. Substance-related disorders
3. Complex trauma

**Objectives:**

Although mental health problems among victims of trafficking have been shown to be high, recovery without treatment is rare, particularly in those who have developed PTSD.

Where there is comorbidity, recovery often does not occur even when rehabilitation has been attempted. This is unsurprising given the multiplicity of trauma that victims of trafficking have experienced, which often includes trauma prior to the trafficking situation.

**Methods:**

Evidence-Based Therapeutic Treatment Options for PTSD:

Some evidence suggests that selective serotonin reuptakeinhibitors can effectively complement the psychotherapeutictreatment of PTSD as well as other anxiety and mooddisorders.

Cognitive-behavioral therapy

Exposure therapy

Eye movement desensitization and reprocessing

Narrative Exposure Therapy (NET)

**Results:**

In the absence of research pertaining to the mental health treatment of victims of human trafficking, mental health professionals working with this population must educate themselves on the evidence based research related to the treatment of common diagnoses and similarly marginalized populations to ensure proper provision of the best mental health care possible.

**Conclusions:**

Among the most devastating mental health consequences for victims of any crime can be the destruction of basic life assumptions; that one is safe from harm, one is a good and decent person, and the world is meaningful and just. For victims of human trafficking, mental health problems can be compounded by the misconceptions about and limited understanding of the issue of human trafficking.

Additionally, lack of social support and stigmatization by friends, family, and social institutions can exacerbate victims mental health conditions.

Psychologically process the trauma they have experienced. However, until these studies are conducted and their results made available, mental health practitioners can base treatment options for this population on existing research findings and interventions found to be successful with other similarly victimized populations.

**Disclosure of Interest:**

None Declared

